# Functional Neuroimaging Correlates of Placebo Response in Patients With Depressive or Anxiety Disorders: A Systematic Review

**DOI:** 10.1093/ijnp/pyac009

**Published:** 2022-01-25

**Authors:** Nathan T M Huneke, Ibrahim H Aslan, Harry Fagan, Naomi Phillips, Rhea Tanna, Samuele Cortese, Matthew Garner, David S Baldwin

**Affiliations:** Clinical and Experimental Sciences, Faculty of Medicine, University of Southampton, Southampton, UK; Southern Health National Health Service Foundation Trust, Southampton, UK; University Department of Psychiatry, Academic Centre, Southampton, UK; Clinical and Experimental Sciences, Faculty of Medicine, University of Southampton, Southampton, UK; University Department of Psychiatry, Academic Centre, Southampton, UK; Southern Health National Health Service Foundation Trust, Southampton, UK; University Department of Psychiatry, Academic Centre, Southampton, UK; Solent National Health Service Trust, Southampton, UK; Southern Health National Health Service Foundation Trust, Southampton, UK; Solent National Health Service Trust, Southampton, UK; Center for Innovation in Mental Health, School of Psychology, Faculty of Environmental and Life Sciences, University of Southampton, Southampton, UK; Hassenfeld Children’s Hospital at NYU Langone, New York University Child Study Center, New York City, New York, USA; Division of Psychiatry and Applied Psychology, School of Medicine, University of Nottingham, Nottingham, UK; Clinical and Experimental Sciences, Faculty of Medicine, University of Southampton, Southampton, UK; School of Psychology, Faculty of Environmental and Life Sciences, University of Southampton, Southampton, UK; Clinical and Experimental Sciences, Faculty of Medicine, University of Southampton, Southampton, UK; Southern Health National Health Service Foundation Trust, Southampton, UK; University Department of Psychiatry, Academic Centre, Southampton, UK; University Department of Psychiatry and Mental Health, University of Cape Town, Cape Town, South Africa

**Keywords:** Placebo response, depression, anxiety, functional neuroimaging

## Abstract

**Background:**

The mechanisms underlying placebo effects of psychotropic drugs remain poorly understood. We carried out the first, to our knowledge, systematic review of functional neuroimaging correlates of placebo response in adults with anxiety/depressive disorders.

**Methods:**

We systematically searched a large set of databases up to February 2021 based on a pre-registered protocol (PROSPERO CRD42019156911). We extracted neuroimaging data related to clinical improvement following placebo or related to placebo mechanisms. We did not perform a meta-analysis due to the small number of included studies and significant heterogeneity in study design and outcome measures.

**Results:**

We found 12 relevant studies for depressive disorders and 4 for anxiety disorders. Activity in the ventral striatum, rostral anterior cingulate cortex and other default mode network regions, orbitofrontal cortex, and dorsolateral prefrontal cortex correlated with placebo antidepressant responses. Activity in regions of the default mode network, including posterior cingulate cortex, was associated with placebo anxiolysis. There was also evidence for possible involvement of the endogenous opioid, dopamine, and serotonin systems in placebo antidepressant and anxiolytic effects.

**Conclusions:**

Several brain regions and molecular systems may be involved in these placebo effects. Further adequately powered studies exploring causality and controlling for confounders are required.

## Introduction

Anxiety and depression are the most common psychiatric conditions (Wittchen et al., 2011) and cause significant distress, impair function, and reduce quality of life. There is a need to improve treatments for these conditions, because many patients do not respond or experience unwanted side effects. Placebo-controlled trials are the gold-standard method for assessing efficacy of medications. However, the placebo response in psychotropic trials is a large effect. Approximately 30% of patients in antidepressant trials demonstrate a placebo response (Walsh et al., 2002; Stein et al., 2006; Furukawa et al., 2016), and in anxiety disorders the effect size of placebo ranges from .65 to 1.29 (Bandelow et al., 2015; De Vries et al., 2016). This has implications for the design and interpretation of psychotropic drug trials. However, the mechanisms underlying placebo effects in depression and anxiety are poorly understood (Huneke et al., 2020).

Symptom improvement in the placebo arm of a trial can be partly explained by nonspecific phenomena, such as regression to the mean or sampling bias due to dropouts of the least improved patients (Ernst and Resch, 1995; Ashar et al., 2017; Evers et al., 2018). However, improvements can also result from specific placebo effects in which an interplay between learning and expectations causes biological changes in the immune system, hypothalamic-pituitary-adrenal axis, and the endogenous opioid system (Ernst and Resch, 1995; Benedetti et al., 2011; Peciña and Zubieta, 2015; Evers et al., 2018). The neuroimaging correlates of placebo effects in particular domains are well understood, such as in placebo analgesia (Atlas and Wager, 2014; Wager and Atlas, 2015; Zunhammer et al., 2021). However, neuroimaging correlates of placebo antidepressant and anxiolytic effects have not been delineated. Identifying these markers might help us understand the mechanisms involved in placebo effects in these conditions. This might allow us to improve clinical trial design or identify novel therapeutic targets (Huneke et al., 2020).

We carried out a systematic review to identify functional neuroimaging correlates of the placebo effect in adults with anxiety or depression. We aimed to understand current knowledge of the neuroanatomy and neurotransmitter systems important in these effects and identify hypotheses to be tested in future studies.

## METHODS

The review was carried out according to PRISMA guidelines (Page et al., 2021). Five authors (N.H., I.A., H.F., N.P., R.T.) performed the systematic review and data extraction independently in pairs. All discrepancies were resolved by consensus. The protocol was registered prospectively with PROSPERO (CRD42019156911).

### Literature Search

Our full search strategy is reported in the supplemental material. We performed the search, with no date or language restrictions, on March 9, 2019, and updated on September 2, 2021. We also reviewed reference lists of relevant review articles for additional records.

At least 2 reviewers screened all titles and abstracts against the following inclusion criteria: the study was a randomized trial involving a placebo intervention; patients were aged 18–65 years with a unipolar depressive or anxiety disorder; patients underwent functional neuroimaging (positron emission tomography [PET], single-photon emission computed tomography, functional magnetic resonance imaging [fMRI]), and change in depressive or anxiety symptoms was an outcome measure. Although not prespecified, we chose to also include arterial spin labelling (ASL) imaging on reviewing our search results to avoid excluding potentially informative studies. We obtained full texts for potentially eligible articles, which were then screened by at least 2 reviewers. Articles were included if they presented neuroimaging data associated with an objective clinical improvement following placebo treatment or with placebo mechanisms such as learning or expectancy.

### Quality Assessment

We assessed for risk of bias with the Cochrane Collaboration’s risk of bias 2 tool for randomized trials (Sterne et al., 2019). One reviewer (I.A., H.F., or R.T.) recorded risk of bias for each record using a standardized form, and these assessments were independently checked by a second reviewer (N.H.). We assessed the risk of bias due to randomization, deviations from the intended intervention, missing data, outcome measurement, and selective reporting.

### Data Extraction and Synthesis

One reviewer (I.A., H.F., or R.T.) extracted data by using a piloted, standardized form. All extracted data were checked independently by a second reviewer (N.H.). We extracted data regarding the patient population, study design, imaging modality, missing data, and key clinical and imaging results.

Due to the small number of included articles, of which only approximately one-half included whole-brain analyses, and the significant heterogeneity in study design and outcome measures, we were unable to conduct a formal meta-analysis. We therefore undertook a narrative synthesis of the data.

## RESULTS

**Figure 1.**
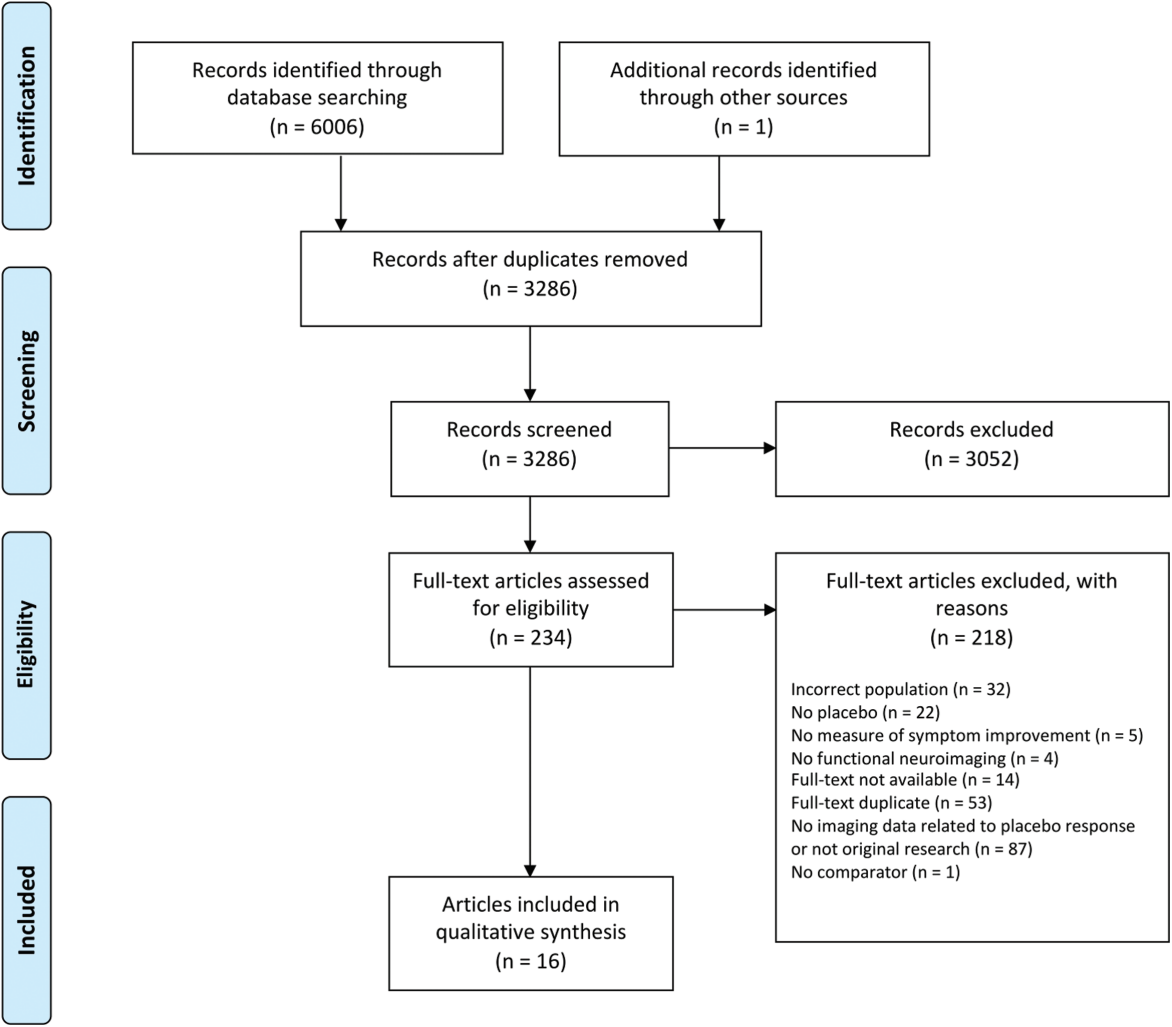
Study selection flow diagram.

Our search initially identified 6006 records. We identified 1 additional record through hand-searching of reference lists. After de-duplication, we screened 3286 titles and abstracts, 234 full-text articles were obtained, and 16 records met inclusion criteria (Figure 1). Twelve studies were of patients with depression utilizing the following imaging modalities: ASL (Cooper et al., 2019), fMRI (Sikora et al., 2016; Pecina et al., 2018; Zilcha-Mano et al., 2019; Chin Fatt et al., 2020; Fan et al., 2020; Greenberg et al., 2020; Chin Fatt et al., 2021b; Chin Fatt et al., 2021a; Peciña et al., 2021), and PET (Mayberg et al., 2002; Pecina et al., 2015). Four studies were in patients with social anxiety disorder (SAD) utilizing PET (Furmark et al., 2008; Faria et al., 2012; Faria et al., 2014) and fMRI (Faria et al., 2017). Sample sizes ranged from 8 to 279 patients, and one-half of the studies were carried out in samples of fewer than 50 patients. There was overlap in these samples with 8 studies of patients with depression (Peciña et al., 2015; Sikora et al., 2016; Cooper et al., 2019; Chin Fatt et al., 2020; Fan et al., 2020; Greenberg et al., 2020; Chin Fatt et al., 2021b; Chin Fatt et al., 2021a) and 3 of patients with SAD (Furmark et al., 2008; Faria et al., 2012, 2014) sharing similar or identical samples. Therefore, the maximum total number of patients included in this review is n = 503. The mean age of the patients in each study ranged from 28.8 years (SD ± 8.6) to 49 years (SD ± 9.0). Most patients were female (67%), with only 2 studies reporting a majority of male patients (Mayberg et al., 2002; Faria et al., 2017).

We report here the key details of the included studies, which are summarized in Tables 1 and 2.

**Table 1.** Summary of Papers Examining Functional Neuroimaging Markers of Placebo Effects in Patients With Depression

Reference	n	Interventions		Study duration	Symptom outcome measure	Imaging modality	Imaging measure	Analysis	Clinical results	Imaging markers of placebo response or placebo mechanisms
		Experimental	Comparator							
Chin Fatt et al. (2020)	279	Sertraline	Placebo	8 wk	HAMD-17	fMRI	Pretreatment resting-state functional connectivity	ROI	Total of 32.2% (90/279) achieved remission (49 sertraline, 41 placebo)	No within-network moderators Higher connectivity of hippocampus with executive control network and lower connectivity of thalamus with visual and salience networks predicted better outcome with placebo Higher connectivity of the hippocampus with the visual, dorsal attention, executive control networks, and thalamus, the limbic network with the salience and somatomotor networks, and the executive control network with the salience and somatomotor networks predicted greater improvement with placebo and worse outcome with sertraline.
Chin Fatt et al. (2021a)	244	Sertraline	Placebo	8 wk	HAMD-17	fMRI	Pretreatment resting-state functional connectivity correlates of subgroups defined through principal component analysis	ROI	Total of 32% (79/244) achieved remission (39 sertraline, 40 placebo)	Subgroups with greater improvement with placebo typified by increased connectivity within the limbic network, between hippocampus and visual network, and salience network with dorsal attention network
Chin Fatt et al. (2021b)	279	Sertraline	Placebo	8 wk	HAMD-17	fMRI	Pretreatment resting-state functional connectivity	ROI	As above	As baseline connectivity between dorsolateral PFC and inferior parietal cortex increased, superiority of sertraline over placebo reduced. This was driven by a relative increase in efficacy of placebo. A similar pattern was seen for baseline connectivity between subcallosal and posterior cingulate cortices.
Cooper et al. (2019)	231	Sertraline	Placebo	8 wk	HAMD-17	ASL	Pretreatment resting-state, relative cerebral perfusion	Whole brain	Total of 35% (81/231) achieved remission: 37% of patients on sertraline vs 33% of patients on placebo	Relative perfusion in right posterior insula; left midbrain; right hippocampus; right inferior frontal; right middle and inferior frontal gyri (including the dorsolateral PFC); left precentral gyrus; left inferior frontal; left middle temporal gyrus; right caudate; left cerebellum; right middle, superior, and inferior frontal gyri; left middle frontal gyrus (and dorsolateral PFC); right middle temporal gyrus; left cuneus; left cingulate; left fusiform gyrus; and the left inferior frontal gyrus moderated response to placebo.
Fan et al. (2020)	200	Sertraline	Placebo	8 wk	HAMD-17	fMRI	Pre-treatment resting-state connectome fingerprints	Whole brain	No significant differences in clinical outcomes between groups	Greater treatment response independent of modality predicted by decreased connectivity between executive, sensorimotor and salience networks, and increased connectivity between default mode network and the rest of the brain. No connectome fingerprint specific to response to either sertraline or placebo was found
Greenberg et al. (2020)	222	Sertraline	Placebo	8 wk	HAMD-17	fMRI	Pre-treatment change in ventral striatal activity during a monetary reward task	ROI	Across groups, HAMD-17 scores significantly improved over time. No separate statistics for each group presented.	Left ventral striatal “reward index” moderated treatment effects. Patients with greater increases in reward activity over time appeared more likely to benefit from placebo. Patients with lower increase in reward expectancy activity over time were more likely to benefit from sertraline.
Mayberg et al. (2002)	8	Fluoxetine	Placebo	6 wk	HAMD-17	PET	Resting-state brain metabolism at baseline and after 6 wk of treatment	Whole brain	Total of 8 of an original 17 responded to treatment (4 placebo, 4 fluoxetine)	Increases seen in PFC (BA 9/46), premotor cortex (BA 6), inferior parietal cortex (BA 40), posterior insula, posterior cingulate (BA 23/31). Decreases seen in sgACC (BA 25), hypothalamus, thalamus, supplementary sensory area, anterior insula, parahippocampus.
Pecina et al. (2015)	35	“Active” placebo	“Inactive” placebo	2 wk	QIDS-SR16	PET	MOR binding potential after “active” vs “inactive” placebo treatment	ROI	Symptom improvement significantly greater for “active” vs “inactive” placebo. Remission at study end significantly higher in placebo responders	Placebo administration reduced MOR binding potential in nucleus accumbens Degree of placebo-induced opioid release in the sgACC, nucleus accumbens, thalamus and amygdala explained 43% of the response to open-label antidepressant treatment.
		Open-label antidepressant	None	10 wk						
Pecina et al. (2018)	20	Placebo IV infusion Positive sham neurofeedback	No infusion	Single session	Subjective expectation of mood improvement and subjective mood trial by trial	fMRI	Change in BOLD signal	Whole brain	Expectancy significantly higher during placebo infusion. Mood significantly improved following placebo infusion, following positive sham neurofeedback, and when expectancy was higher.	Positive sham neurofeedback led to greater activity in bilateral ventro- and dorsolateral PFC, which was positively correlated with improved mood. Increased activity in left ventro- and dorsolateral PFC associated with greater expectancy when mood was rated higher in previous trial. However, activity in bilateral ventro- and dorsolateral PFC also negatively moderated the effect of higher expectation on subsequent mood improvement.
			Negative sham neurofeedback							
Pecina et al. (2021)	20	Naltrexone 50 mg Placebo IV infusion Positive sham neurofeedback	Placebo No infusion Negative sham neurofeedback	7–10 d	Subjective expectation of mood improvement and subjective mood trial by trial	fMRI	Change in BOLD signal	Whole brain	Expectancy significantly higher during placebo infusion. Mood significantly improved following positive sham neurofeedback, and this was greater when expectancy was higher.	Higher activity in right ventro- and dorsolateral PFC associated with reduced expectancy*reinforcement condition effect on expectancy and mood ratings.
										Naltrexone partially abolished the expectancy*reinforcement condition effect on expectancy and mood ratings. This was associated with reduced responses in right OFC during processing of positive reinforcement. Participants with greater naltrexone-induced modulation of OFC activity during positive sham neurofeedback had higher expectancy and mood ratings
Sikora et al. (2016)	29	“Active” placebo	“Inactive” placebo	2 wk	QIDS-SR16	fMRI	Resting-state functional connectivity after “active” and “inactive” placebo	Whole-brain and exploratory ROI	Symptom improvement significantly greater for “active” vs “inactive” placebo.	Increased “baseline” connectivity of the rACC with the salience network was significantly associated with greater placebo response. Placebo-induced reduction in rACC with the salience network was also associated with greater placebo response. “Baseline” resting-state connectivity of the salience network was significantly predictive of placebo response.
		Open-label antidepressant	None	10 wk						
Zilcha-Mano et al. (2019)	23	High expectation (100% chance of receiving citalopram)	Low expectation (50% chance of receiving citalopram)	1 wk	HAMD-24	fMRI	Change in BOLD signal during a masked emotional face task at baseline and 1 wk after randomization to high or low expectation	Whole-brain and ROI	Patients in the high expectation group demonstrated significantly greater outcome expectation.	High expectation group showed a decrease in amygdala activation from scan 1 to 2 in the sad vs neutral face contrast, whereas low expectation group showed an increase. Increases in outcome expectancy significantly correlated with reductions in left amygdala activity. Total of 63.41% of the effect of outcome expectancy on change in HAMD-24 was mediated by changes in amygdala activity.
		Citalopram	Placebo	8 wk						

Abbreviations: ASL, Arterial spin labelling; BA, Brodmann area; BOLD, blood-oxygen-level-dependent imaging; fMRI, functional magnetic resonance imaging; HAMD, Hamilton rating scale for depression; MOR, mu-opioid receptor; OFC, orbitofrontal cortex; PET, positron emission tomography; PFC, prefrontal cortex; QIDS-SR, Quick inventory of depressive symptomatology (self-report); rACC, rostral anterior cingulate cortex; ROI, region of interest; sgACC, subgenual anterior cingulate cortex.

**Table 2.** Summary of papers examining functional neuroimaging markers of placebo effects in patients with social anxiety disorder

Reference	n	Interventions		Study duration	Treatment response definition	Imaging modality	Imaging measure	Clinical results	Imaging markers of placebo response or placebo mechanisms
		Experimental	Comparison						
Faria et al. (2012)	72	Citalopram or paroxetine	Placebo	6-8 wk	CGI-I = 1 or 2	PET	rCBF during a public speaking task at baseline and study end	Total of 57% of SSRI group responded vs 30% of placebo group	Both SSRI and placebo responders showed reductions in right ventrolateral amygdala and left basomedial/basolateral amygdala. The rCBF change correlated with clinical measures of anxiety. Placebo responders additionally showed increased rCBF in right brainstem/pons compared with placebo nonresponders.
Faria et al. (2014)	72	Citalopram or paroxetine	Placebo	6-8 wk	CGI-I = 1 or 2	PET	Functional connectivity during a public speaking task at baseline and study end	Total of 57% of SSRI group responded vs 30% of placebo group	Placebo responders showed greater negative correlation between left amygdala and left dorsolateral PFC vs placebo nonresponders. Placebo responders showed greater negative correlation between left amygdala and right ventromedial and dorsolateral PFC, and greater positive correlation between left amygdala and right dorsomedial PFC vs SSRI responders.
Faria et al. (2017)	46	“Overt” escitalopram	“Covert” escitalopram	9 wk	LSAS-SR < 39	fMRI	BOLD signal change and functional connectivity during emotional face matching task	“Overt” treatment significantly superior (*d* = .24 vs *d* = 1.13)	Increased reactivity to emotional faces in overt vs covert in bilateral posterior cingulate, left mid temporal gyrus, left inferior frontal gyrus. Covert group showed increased connectivity between amygdala and right posterior cingulate and right insula when viewing emotional faces compared with overt group.
Furmark et al. (2008)	25	Placebo	None	8 wk	CGI-I = 1 or 2	PET	rCBF during a public speaking task at baseline and study end. Changes in rCBF associated with genotype.	Total of 10 (40%) responded to placebo	rCBF significantly reduced in left amygdala in placebo responders vs nonresponders. Patients homozygous for the long allele of 5-HTTLPR and/or the G allele of the G-703T polymorphism in TPH2 exhibited a significantly greater reduction in amygdala activity vs heterozygotes. Mediation analysis showed that the change in rCBF in the amygdala mediated the effect of G-703T polymorphism on CGI-I score.

Abbreviations: 5-HTTLPR, serotonin transporter-linked polymorphic region; BOLD, blood-oxygen-level-dependent imaging; CGI-I, Clinical Global Impression-Improvement scale; fMRI, functional magnetic resonance imaging; LSAS-SR, Liebowitz Social Anxiety scale; PET, positron emission tomography; PFC, prefrontal cortex; rCBF, regional cerebral blood flow; SSRI, selective serotonin reuptake inhibitor; TPH2, tryptophan hydroxylase-2.

### Depression

Twelve studies described imaging markers of placebo antidepressant responses. The first published study to report imaging markers of placebo antidepressant responses was carried out by Mayberg et al. (2002). In this 6-week randomized trial of fluoxetine compared with placebo, 8 of 15 patients with depression responded to treatment (fluoxetine n = 4, placebo n = 4). The patients underwent PET imaging at baseline and at 1 week and 6 weeks after commencing treatment, and changes in regional cerebral glucose metabolism at these timepoints were computed separately for the placebo and drug responder groups. Placebo response was associated with significant regional changes in metabolism (beta-2_(1972) _=_ _3.97, *P* < .0001). Increased cerebral glucose metabolism was seen in regions including dorsolateral prefrontal cortex, posterior insula and posterior cingulate cortex; and decreased metabolism in subgenual anterior cingulate cortex, thalamus, anterior insula, and parahippocampus. These regions overlapped with those seen in patients who responded to fluoxetine.

Six relevant papers for this review have been published from the Establishing Moderators and Biosignatures of Antidepressant Response in Clinical Care (EMBARC) trial (Cooper et al., 2019; Chin Fatt et al., 2020, 2021a, 2021b; Fan et al., 2020; Greenberg et al., 2020). The aim of this trial was to identify neural predictors or correlates of response to treatment. Patients with major depressive disorder (n = 296) underwent ASL and fMRI at rest, and fMRI during a monetary reward task, before randomization to treatment with either sertraline or placebo. The 17-item Hamilton Rating Scale for Depression (HAMD-17) was used to monitor response to treatment.

Of the original 296 patients, 231 completed the baseline ASL scan. Of these, 37% of patients who received sertraline and 33% of patients who received placebo achieved remission (HAMD-17 < 7). Through a whole-brain, voxel-wise, linear mixed-effects model of the ASL and clinical data, 30 clusters of >100 voxels were found to be significant moderators of treatment response, that is, these brain regions showed a significant treatment×time×relative cerebral perfusion interaction. Perfusion in regions including right putamen and insula, left inferior temporal gyrus, right orbital frontal gyrus, and left parahippocampal gyrus moderated response to sertraline. Conversely, moderators of placebo response included regions involved in cognitive control and the default mode networks, such as right posterior insula, right orbital frontal cortex, and right dorsolateral prefrontal cortex (Cooper et al., 2019).

A number of analyses of the baseline resting-state fMRI data have been conducted (Chin Fatt et al., 2020, 2021a, 2021b; Fan et al., 2020). In an analysis exploring pretreatment resting-state connectome fingerprints of treatment response in 200 of the EMBARC patients, no connectome fingerprints specific to placebo response were found (Fan et al., 2020). In a larger sample of 244 patients, connectivity correlates of subgroups identified through principal component analysis were explored. Increased connectivity within the limbic network, between hippocampus and visual network, and salience network with dorsal attention network was associated with subgroups who experienced greater improvement with placebo (Chin Fatt et al., 2021a). Finally, 2 analyses were conducted in 279 EMBARC patients. The first was an exploratory seed-based analysis of 7 networks and some midbrain regions including hippocampus, striatum, thalamus, and amygdala, in a moderation model. Higher connectivity of the hippocampus with the thalamus and the visual, dorsal attention, and executive control networks, and the limbic and the executive control networks with the salience and somatomotor networks predicted improved outcomes with placebo and worse outcomes with sertraline (Chin Fatt et al., 2020). In the second analysis, connectivity between 5-mm-sphere seeds of interest thought to be within the same functional network was included as a term in a moderation analysis. As baseline connectivity between dorsolateral prefrontal cortex and inferior parietal cortex increased, the superiority of sertraline over placebo treatment reduced (*P* = .05). The reduced superiority of sertraline was driven by a relative increase in efficacy of placebo (Chin Fatt et al., 2021b). The differing sample sizes in these analyses were not fully explained, and so there is a risk of bias in these findings.

Finally, 222 EMBARC patients completed a monetary reward task at baseline (Greenberg et al., 2020) to identify whether temporal changes in reward processing within the ventral striatum predicted or moderated treatment response. The authors calculated a “reward index” from the sum of the increase in reward expectancy and the decrease in prediction error-related activity in the ventral striatum from the first half to the second half of the task. The left ventral striatal reward index significantly moderated treatment effects (F_(1,193)_ =_ _12.93, *P* = .0004). Reduced left ventral striatal reward index at baseline conferred greater likelihood of deriving benefit from treatment with sertraline compared with placebo (threshold Z = −.21, raw HAMD-17 difference of ≥3, t_(193) _=_ _2.38, *P* = .02, *d* = .32, 95% CI = 0.06 to 0.58). The threshold at which patients were expected to benefit more greatly from placebo was not directly tested, but from data presented in the paper, when reward index Z > 2, placebo treatment showed an advantage of approximately 0 to 4 points on the HAMD-17 over sertraline (Greenberg et al., 2020).

In a version of the “open-hidden” paradigm, Zilcha-Mano et al. (2019) explored the neural correlates of expectancy augmentation in an antidepressant trial. Twenty-three patients with depression underwent fMRI scanning while they viewed masked emotional faces displaying fearful, sad, happy, or neutral expressions. The patients were then randomized to 1 of 2 groups: an open-label group that had 100% chance of receiving citalopram (n = 9) or a placebo-controlled group that had a 50% chance of receiving either citalopram or placebo (n = 14). One week later, after being told which group they had been allocated to but before they received treatment, patients underwent a second fMRI scan while completing the same emotional face task. Following this, the patients completed an 8-week clinical trial of citalopram compared with placebo. Patients in the open group showed significantly improved outcome expectancy post-randomization compared with the placebo-controlled group (W = 31.5, *P* = .007). Further, the open-label group showed a significant reduction in activity in the amygdala, bilateral dorsolateral prefrontal cortex, and superior temporal gyrus following randomization compared with the placebo-controlled group in the sad vs neutral faces contrast. The amygdala was then chosen as a region of interest, and a linear association was found between reduction in left amygdala activity and increase in expectancy score post-randomization (r = −.74, *P* = .006). A mediation analysis showed that HAMD-24 scores decreased at a faster rate for patients with increased expectancy scores, and this was mediated by greater reductions in amygdala activity post-randomization (B = −.09, *P* = .007). However, we noted possible selective reporting in this trial. The amygdala only showed a significant difference in activity in the sad vs neutral faces contrast, whereas other regions demonstrated significant differences in activity in other relevant contrasts. The amygdala is then chosen as a region of interest with little justification, and no further analyses regarding other significant regions are reported (Zilcha-Mano et al., 2019).

Peciña et al. (2015) and Sikora et al. (2016) explored whether imaging correlates of placebo mechanisms can predict antidepressant treatment outcomes using an experimental placebo lead-in phase followed by a 10-week open-label antidepressant trial. Patients were given oral placebo with instructions that this was an antidepressant for 1 week (“active”) followed by a 3-day washout and then 1 week of treatment with “inactive” placebo, with disclosure that this was an inert control. After each placebo condition, participants underwent neuroimaging. Results from PET imaging with the µ-opioid receptor-selective radiotracer [^11^C]carfentanil were reported in 35 patients. After the “active” placebo condition, the PET session additionally included an i.v. infusion of .9% isotonic saline with instructions this was a “rapid-acting antidepressant” as an acute placebo challenge to induce endogenous opioid release. Placebo administration during the PET scan reduced µ-opioid receptor binding potential in the nucleus accumbens (estimate = −.43, Z = 4.72, *P* < .001). Further, degree of placebo-induced opioid release in the subgenual anterior cingulate cortex, nucleus accumens, thamalus, and amygdala was associated with reduction in depressive symptoms after 1 week of “active” placebo (estimates ≤ −.38, Z > 3.80, *P* < .001) and with response to open-label antidepressant at 10 weeks (estimates ≤ −.60, Z > 3.98, *P* < .001). Results from resting-state fMRI scans after each placebo condition were reported in 29 patients. Reduction in depressive symptoms was significantly greater after 1 week of the “active” placebo than after “inactive” placebo (F = 7.2, *P* = .012). Increased baseline resting functional connectivity (Z = 4.35, adjust R^2^ = .65, *P* < .005) and reduction in connectivity following “active” placebo of the rostral anterior cingulate cortex within the salience network (Z = 3.97, *P* < .05) were associated with greater placebo response (Sikora et al., 2016). However, we identified some potential risks of bias. First, the “baseline” was the scan carried out after 1 week of “inactive” placebo. This does not represent a true baseline due to the crossover design. Placebo analgesia is reduced if participants have experienced a previously ineffective analgesic treatment (Colloca and Benedetti, 2006). Such effects could potentially confound these results. Second, no explanation is given for the discrepancy in sample size in these papers.

Two studies by Pecina et al. (2018, 2021) attempted to manipulate trial-by-trial antidepressant expectancies through a “simulated neurofeedback” task. In brief, this task involved 6 runs of 12 trials, where each trial began with a timer cue reflecting an anticipation period prior to either receiving or not receiving a “rapid-acting antidepressant” infusion (in reality, normal saline). After the infusion cue, participants were shown sham neurofeedback with differing valence (either positive or negative). After both the anticipation and simulated neurofeedback periods, participants rated their expected and actual mood improvements, respectively. In the 2018 study involving 20 patients, there was greater mood improvement during the infusion cue (b = .12, *P* < .05) and following the display of positive sham neurofeedback (b = .32, *P* < .001), and higher expectation of benefit predicted improved mood (b = .22, *P* < .001). Positive sham neurofeedback led to greater activity in bilateral ventro- and dorsolateral prefrontal cortices, which was positively correlated with improved mood (b = .2, *P* < .001). Increased activity in left ventro- and dorsolateral prefrontal cortices was also associated with greater expectancy when mood improved in the previous trial (b = .05, *P* < .05). However, activity in bilateral ventro- and dorsolateral prefrontal cortices also negatively moderated the effect of higher expectation on subsequent mood improvement (b = −.07, *P* < .05). Finally, β-endorphin plasma levels were also measured before and after the task. Greater increases in β-endorphins were associated with increased expectancy ratings (estimate = .0007, *P* = .02) and greater subjective mood improvement in response to positive neurofeedback (estimate = .002, *P* < .001) (Peciña et al., 2018). In a subsequent double-blind crossover study, 20 patients with depression carried out the same neurofeedback task twice: once following treatment with naltrexone 50 mg and once following matched placebo. In this study, higher activity in the right ventro- and dorsolateral prefrontal cortex was again associated with a reduced placebo×neurofeedback condition effect on expectancy and mood ratings. Naltrexone reduced the effect of the placebo×neurofeedback condition interaction on expectancy (b = −1.00, *P* < .001) and mood ratings (b = −.93, *P* = .003). Naltrexone was also associated with reduced activity in the right orbitofrontal cortex during positive sham neurofeedback (max t = 5.64, cluster size = 334 voxels, *P* < .001). Greater naltrexone-induced reductions in orbitofrontal cortex activity during positive sham neurofeedback correlated with higher expectancy during the “antidepressant” condition (b = .40, *P* < .01) (Peciña et al., 2021).

### Social Anxiety Disorder

Four studies involving patients with SAD were included in this review. First, in a pooled secondary analysis of 2 randomized-controlled trials, 25 patients with SAD completed a public speaking task while undergoing PET imaging before and after 8 weeks of placebo treatment. The patients’ genotypes for the serotonin transporter-linked polymorphic region (5-HTTLPR) and the tryptophan hydroxylase-2 (TPH2) gene promoter were also obtained. Ten of the 25 patients (40%) were classified as placebo responders based on reduction in clinical global impression scale at study end. Regional cerebral blood flow in the left amygdala during the public speaking task decreased significantly more in placebo responders compared with nonresponders (Z = 2.64, *P* = .048). There was an additional effect of genotype in that only patients homozygous for the long allele of 5-HTTLPR and/or the G allele of the G-703T polymorphism in TPH2 exhibited a placebo response. A mediation analysis suggested that reduction in amygdala activity mediated the effect of the TPH2 polymorphism on placebo response (*P* = .029) (Furmark et al., 2008). These results raise the possibility that the amygdala and serotonin are important in placebo anxiolytic responses. However, this was a small sample size for this analysis, so there is a risk of false positives.

In a subsequent study, Faria et al. (2012, 2014) aimed to delineate the roles of different amygdala subregions in placebo anxiolysis. The 72 patients included in this study were pooled from 3 placebo-controlled trials of citalopram or paroxetine vs placebo, some of whom were also in the sample described above (Furmark et al., 2008), again undergoing PET imaging during a public speaking task before and after treatment. Twenty of 35 patients were classified as selective serotonin reuptake inhibitor (SSRI) responders (57%), and 11 of 37 patients responded to placebo (30%) (Faria et al., 2012). All treatment responders exhibited reduced cerebral blood flow in the left basomedial/basolateral (Z = 2.49, *P* < .005) and right ventrolateral amygdala (Z = 2.95, *P* < .05) subregions during the second PET scan. Moreover, the change in blood flow in these regions correlated significantly with reduced anxiety (r > 0.3, *P* < .005). There were no differences between SSRI and placebo responders (Faria et al., 2012). An analysis of functional connectivity patterns in these patients showed that placebo responders exhibited increased negative correlation between left basomedial/basolateral amygdala activity and left dorsolateral prefrontal cortex compared with nonresponders (Z = 3.42, *P* < .001). Compared with SSRI responders, placebo responders showed greater negative correlation between left amygdala and right ventromedial and dorsolateral prefrontal cortices and more positive correlation with dorsomedial prefrontal cortex (Z > 3.00, *P* = .001) (Faria et al., 2014). Inferences regarding potential neurotransmitters involved cannot be made from these data.

Finally, the role of expectations in augmenting antidepressant treatment was explored in 46 patients (Faria et al., 2017). All were treated with escitalopram for 9 weeks but were randomized regarding their instructions. Prior to treatment, 24 patients were informed that they would receive escitalopram while 22 patients were told they would receive an “active placebo” likely to induce side-effects like escitalopram but with no expected symptom improvement. At the beginning and end of treatment, these patients underwent fMRI scanning while they completed an emotional face-matching task. Overt escitalopram treatment caused significantly improved outcomes on the Liebowitz social anxiety scale (*d* = 2.24 vs *d* = 1.13 for covert treatment). The fMRI results showed that overt treatment was associated with increased activity to emotional faces in the bilateral posterior cingulate cortex, left mid temporal gyrus, and left inferior frontal gyrus compared with covert treatment at the end of the study (Z > 3.60, *P* ≤ .0001). A psychophysiological interaction analysis demonstrated that the covert arm exhibited increased connectivity relative to the overt arm between the amygdala and right dorsal posterior cingulate cortex, and right insula, when viewing faces compared with shapes (Z ≥ 2.85, *P* ≤ .002). This was interpreted by the authors as evidence of greater fear processing in the covert group. However, there was no statistically significant difference in amygdala reactivity between groups when viewing emotional faces.

### Results of Quality Assessment

**Figure 2.**
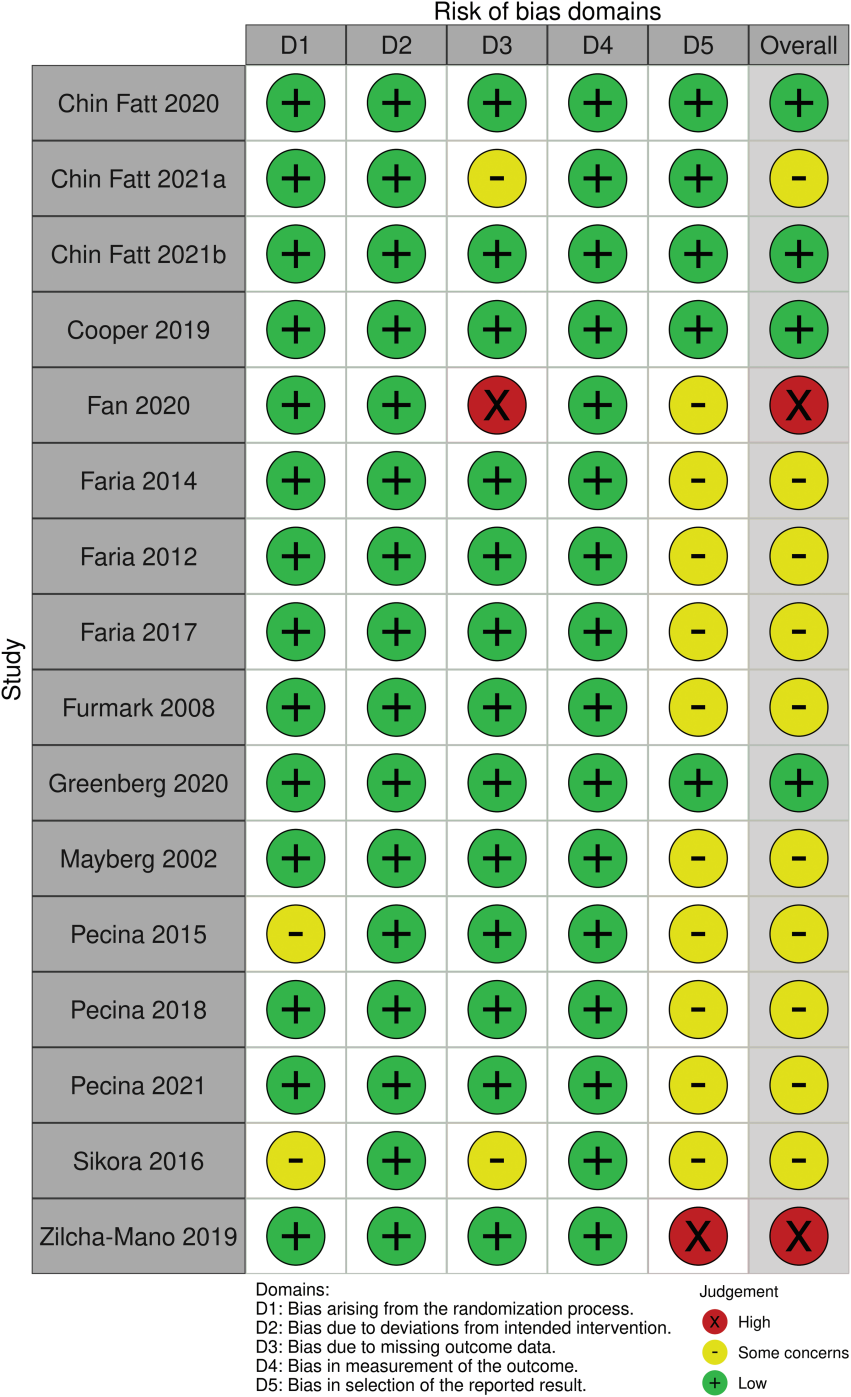
Traffic light plot summarizing review authors’ judgements regarding risk of bias for each included study.

**Figure 3.**
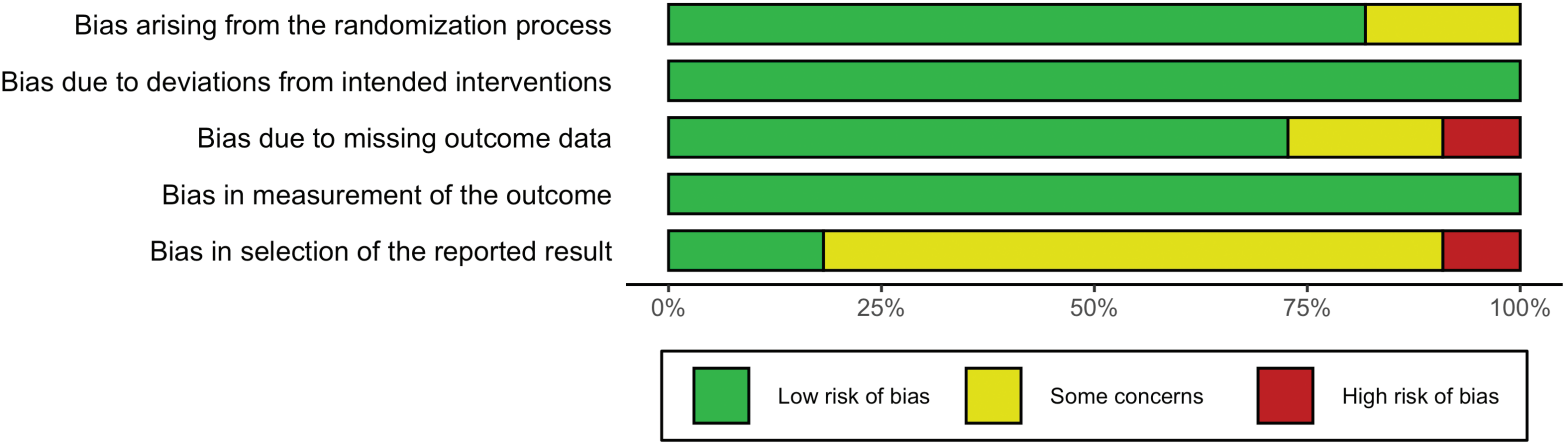
Plot showing review authors’ judgements regarding risk of bias by percentage.

The results of our quality assessment are summarized in Figures 2 and 3. Most studies (10, 62.5%) were rated as “some concerns.” For most, this was due to the lack of a preregistered analysis plan, which meant practices such as dichotomizing treatment groups or the use of “small volume correction” might represent selective reporting (Mayberg et al., 2002; Furmark et al., 2008; Faria et al., 2012, 2014, 2017; Peciña et al., 2015, 2018, 2021; Sikora et al., 2016). In addition, we had concerns regarding missing data for 3 studies (Pecina et al., 2015; Sikora et al., 2016; Chin Fatt et al., 2021a). Two studies were rated as high risk of bias: Fan et al. (2020) due to missing data as results are reported for only 200 participants from the EMBARC trial (as opposed to the 296 reported in other papers) with no justification for this difference; and Zilcha-Mano et al. (2019) due to apparent selective reporting of results as detailed above. See supplemental material for full details of how each risk of bias rating was reached.

## DISCUSSION

To our knowledge, this is the first systematic review of the functional neuroanatomy of placebo response in patients with anxiety or depression. We identified 12 articles reporting functional neuroimaging markers of placebo antidepressant responses and 4 reporting markers of placebo anxiolytic responses. There was substantial heterogeneity in terms of sample size, imaging modality, whether patients were imaged at rest or during a task, at baseline or longitudinally, the type of task, and the primary aim of the study. Further, coordinates of peak activity related to placebo responses were not consistently reported. We instead relied on authors’ naming of brain regions, possibly introducing further inter-study variation. It is therefore challenging to coherently synthesize the data to identify relevant patterns. Nevertheless, there are signals suggesting possible neuroanatomical correlates of, and important neurotransmitter systems in, placebo antidepressant and anxiolytic effects.

### Functional Neuroanatomical Correlates of Placebo Antidepressant and Anxiolytic Effects

Data from the EMBARC trial suggest that the ventral striatum (VS) might be important in placebo antidepressant effects. Reduced reward-related activity in the VS suggested patients were more likely to benefit from sertraline. Conversely, increased activity suggested no advantage of medication and a trend towards superiority of placebo (Greenberg et al., 2020). Significant superiority for placebo would likely be difficult to demonstrate in such a comparison because placebo effects operate in the medication arm (Huneke et al., 2020) as well as other nonspecific effects such as regression to the mean. A role for the VS in placebo antidepressant effects is further supported by the finding that “active” placebo treatment is associated with increased opioid release in the nucleus accumbens compared with an “inactive” placebo (Peciña et al., 2015). The VS is activated by placebo analgesia (Atlas and Wager, 2014), suggesting that reward circuitry might be important in placebo effects across domains.

Activity in dorsolateral prefrontal cortex (dlPFC) correlated with placebo response in many studies (Mayberg et al., 2002; Faria et al., 2014; Peciña et al., 2018, 2021; Cooper et al., 2019; Chin Fatt et al., 2020, 2021b; Fan et al., 2020). This region is reliably activated by placebo analgesia and is thought to be important in generating placebo-related expectancies (Atlas and Wager, 2014; Wager and Atlas, 2015). From the current data, activity increased in the dlPFC in placebo antidepressant responders after 6 weeks (Mayberg et al., 2002), and baseline blood flow in this region moderated subsequent placebo response (Cooper et al., 2019). In placebo responders with SAD, there was decreased correlation between bilateral amygdala and dorsolateral prefrontal cortices during a public speaking task compared with SSRI responders (Faria et al., 2014). Importantly, expectation of mood improvement led to greater dorsolateral/ventrolateral prefrontal cortex activity (Peciña et al., 2018, 2021). This suggests that lateral prefrontal cortex is important in maintaining antidepressant expectancies. However, activity here also negatively moderated the effect of higher expectation and positive reinforcement on subsequent mood improvement (Peciña et al., 2018, 2021). It is possible this finding was due to a “ceiling” effect, because lateral prefrontal cortex was activated when mood had already improved (Peciña et al., 2018). A recent meta-analysis found that placebo analgesia-induced activation of the dlPFC varies greatly between studies (Zunhammer et al., 2021), further making its role in placebo effects difficult to interpret.

Activity in the rostral anterior cingulate cortex (rACC) was identified as important by only 1 study in this review (Sikora et al., 2016). The rACC is activated in placebo analgesia (Atlas and Wager, 2014) and in placebo anxiolysis in healthy volunteers (Petrovic et al., 2005; Meyer et al., 2019). The rACC is in the default mode network, and this network is potentially crucial in generating placebo effects (Ashar et al., 2017). Indeed, placebo response was correlated with increased activity in regions within the default mode network in a number of studies (Mayberg et al., 2002; Faria et al., 2017; Cooper et al., 2019). Further studies are needed investigating the role of the default mode network in placebo antidepressant or anxiolytic effects.

Orbitofrontal cortex (OFC) activity was identified by a single study (Peciña et al., 2021). Placebo analgesia correlates with increased activity in centro-lateral OFC (Wager and Atlas, 2015; Ashar et al., 2017), and this region is densely populated with µ-opioid receptors (Van Steenbergen et al., 2019). The OFC is considered to be important in judging value and encoding expectations regarding outcomes or future events (Wager and Atlas, 2015; Van Steenbergen et al., 2019). Consistently, when µ-opioid receptors were blocked by naltrexone, antidepressant expectancies and the effects of positive reinforcement on mood were reduced, and this was associated with reduced right central orbitofrontal cortex activity (Peciña et al., 2021).

Activity in the amygdala was correlated with placebo anxiolytic and antidepressant effects in a number of studies (Furmark et al., 2008; Faria et al., 2012, 2014, 2017; Peciña et al., 2015; Zilcha-Mano et al., 2019). Three of these studies involved an overlapping sample of patients with SAD, so the reduction in amygdala activity seen could be considered a single finding (Furmark et al., 2008; Faria et al., 2012, 2014). In the fourth study of placebo anxiolysis, there was no evidence of a significant difference in amygdala activity between “overt” and “covert” SSRI administration (Faria et al., 2017). Change in amygdala activity instead correlated with improvement in social anxiety symptoms rather than expectations (Faria et al., 2017). It is therefore unclear whether changes in amygdala activity are due to placebo mechanisms or represent a non-specific phenomenon. Determining this is difficult owing to no “no treatment” arms for comparison, although such arms can be problematic in themselves (Huneke et al., 2020). The 2 studies involving patients with depression carried out functional neuroimaging prior to administration of any active medication (Peciña et al., 2015; Zilcha-Mano et al., 2019). Both studies showed that increased expectation of benefit related to either reduced activity or increased opioid binding in the amygdala (Peciña et al., 2015; Zilcha-Mano et al., 2019). However, both studies were judged to be at risk of bias due to lack of blinding (Peciña et al., 2015) and selective reporting of outcomes (Zilcha-Mano et al., 2019). Placebo and expectancy-induced reductions in bilateral amygdala activity have been found during placebo analgesia (Atlas and Wager, 2014) and in association with reduced feelings of “unpleasantness” when viewing aversive pictures (Petrovic et al., 2005). In the latter study, this did not correlate with placebo response (Petrovic et al., 2005). Further, the large EMBARC trial did not find a relationship between blood flow in the amygdala and placebo response (Cooper et al., 2019). Subgroups responsive to placebo in this trial did have increased resting connectivity within the limbic network (including bilateral amygdala) at baseline (Chin Fatt et al., 2021a); however, when looking at predictors of placebo response alone and not predictors of worse outcomes with sertraline, there was no evidence of amygdala involvement (Chin Fatt et al., 2020). It is possible instead that reductions in amygdala activity represent a phenomenon nonspecific to placebo, perhaps relating instead to treatment response or changes in affect. This needs further exploration.

### Possible Neurotransmitter Systems Involved in Placebo Antidepressant and Anxiolytic Effects

The current data show direct evidence only for a role of the endogenous opioid system in placebo antidepressant effects. A placebo antidepressant caused opioid release in the nucleus accumbens (Peciña et al., 2015), and the administration of naltrexone reduced the effects of expectancy and learning on antidepressant placebo effects (Peciña et al., 2021). There was also additional indirect evidence: increased expectation of benefit and higher mood ratings from a placebo antidepressant were associated with greater increases in plasma β-endorphin levels (Peciña et al., 2018). The endogenous opioid system is important in placebo analgesia (Fields, 2004; Benedetti et al., 2011). Although the present data are limited, they suggest endogenous opioids might be important in placebo effects in other domains, including those involving affect. This is supported by studies of placebo anxiolysis in healthy volunteers, which show overlap with regions important in placebo analgesia (Petrovic et al., 2005; Meyer et al., 2019).

There was further indirect evidence for a role of dopamine in placebo antidepressant effects. The VS was identified as a neuroanatomical correlate of placebo antidepressant effects in 2 studies (Peciña et al., 2015; Greenberg et al., 2020). The VS is also reliably activated in placebo analgesia (Atlas and Wager, 2014) and is an important center of dopaminergic neurotransmission. There is direct evidence for dopamine mediating placebo effects in other domains, including pain (Scott et al., 2008) and Parkinson’s disease (De La Fuente-Fernandez, 2001; Lidstone et al., 2010). Further work is required to understand whether dopamine plays a mediating role in placebo antidepressant effects.

Finally, 1 study in this review showed indirect evidence for a role for serotonin in placebo anxiolytic effects. Presence of the G allele of the G-703T polymorphism in TPH2 mediated placebo-induced reduction in CGI-I score in patients with SAD via a reduction in amygdala activity. As discussed above, it is unclear whether this result is specific to placebo effects or whether this represents another non-specific treatment effect. Furthermore, this analysis involved a small sample size and so there is a possibility this is a false positive. There is no other evidence to our knowledge that serotonin plays a role in placebo anxiolysis or in other placebo effects.

### Limitations

This review has some limitations. First, as with all systematic reviews, we are limited by the quality of the component studies we included. The results of our quality analysis suggest that there was potential for false positives and selective reporting. Where applicable, those findings should be considered with caution. Second, we did not carry out formal meta-analysis for several reasons: the small number of included studies, substantial overlap of study samples, a small number of whole-brain analyses, and the considerable heterogeneity between studies. Therefore, we can only make limited inferences about the relative importance of findings between studies.

## CONCLUSION

We carried out the first systematic review of functional neuroimaging correlates of placebo response in patients with depressive or anxiety disorders. Although limited by the heterogeneity of the studies included in this review, our results suggest that activity in the rACC and default mode network, the VS, OFC, and dlPFC might be central in placebo antidepressant and anxiolytic effects. These regions’ role in causing these effects is less certain and needs further investigation. Meanwhile activity in the amygdala might represent a nonspecific treatment effect. Important neurotransmitter systems could include the endogenous opioid system, dopamine, and serotonin. These hypotheses need further exploration in adequately powered studies designed with the primary aim of exploring the placebo effect, with consideration to possible confounds such as order effects, and involving longitudinal neuroimaging to begin to unpick causality.

## Supplementary Material

pyac009_suppl_Supplementary_Material

pyac009_suppl_Supplementary_Data

## Data Availability

Data accompanying this systematic review are available on the Open Science Framework (https://osf.io/fvb3a/).

## Interest Statement

N.T.M.H. has consulted for Emteq Ltd with no compensation. All other authors declare no potential conflict of interest.
