# Supplementary material for: Functional Neuroimaging Correlates of Placebo Response in Patients With Depressive or Anxiety Disorders: A Systematic Review
Source: Int J Neuropsychopharmacol. 2022 Jan 25;25(6):433–47. doi: 10.1093/ijnp/pyac009 (PMC9211006; doi:10.1093/ijnp/pyac009)
Supplement: pyac009_suppl_Supplementary_Material [file pyac009_suppl_supplementary_material.doc]

**SEARCH STRATEGY AND RESULTS FROM EACH DATABASE**

**PUBMED (MEDLINE)**

- Search terms:

(Placebo OR placebos OR placebo effect OR "placebo response" OR nocebo OR nocebos) AND (Depression OR depressive OR Anxiety OR anxious OR major depressive disorder OR MDD OR generalized anxiety disorder OR GAD OR panic disorder OR simple phobia OR antidepressant OR fluoxetine OR Citalopram OR Escitalopram OR Fluvoxamine OR Paroxetine OR Sertraline OR anxiolytic OR anxiolytics OR benzodiazepine OR benzodiazepines OR alprazolam OR clonazepam OR diazepam OR lorazepam OR temazepam) AND (positron emission tomography OR PET OR Single-photon emission computed tomography OR SPECT OR functional magnetic resonance imaging OR functional MRI OR fMRI OR tomography OR cerebral blood flow)

Used by [*(placebo OR placebos OR placebo effect OR "placebo response" OR nocebo OR****nocebo****) AND (depression OR depressive OR anxiety OR anxious OR major depressive disorder OR MDD OR generalized anxiety disorder OR GAD OR panic disorder OR simple phobia OR antidepressant OR fluoxetine OR citalopram OR escitalopram OR fluvoxamine OR paroxetine OR sertraline OR anxiolytic OR anxiolytics OR benzodiazepine OR benzodiazepines OR alprazolam OR clonazepam OR diazepam OR lorazepam OR temazepam) AND (positron emission tomography OR PET OR single-photon emission computed tomography OR spect OR functional magnetic resonance imaging OR functional mri OR fmri OR tomography OR cerebral blood flow)*](https://www.ncbi.nlm.nih.gov/pubmed?term=(placebo OR placebos OR placebo effect OR "placebo response" OR nocebo OR nocebo) AND (depression OR depressive OR anxiety OR anxious OR major depressive disorder OR MDD OR generalized anxiety disorder OR GAD OR panic disorder OR simple phobia OR antidepressant OR fluoxetine OR citalopram OR escitalopram OR fluvoxamine OR paroxetine OR sertraline OR anxiolytic OR anxiolytics OR benzodiazepine OR benzodiazepines OR alprazolam OR clonazepam OR diazepam OR lorazepam OR temazepam) AND (positron emission tomography OR PET OR single-photon emission computed tomography OR spect OR functional magnetic resonance imaging OR functional mri OR fmri OR tomography OR cerebral blood flow)&cmd=correctspelling)

Limits: **none**

Results: 876 (+ 114 in updated search) hits

**OVID databases**

**PsycInfo, EMBASE+EMBASE classic, OVID Medline**

Search terms:

(Placebo OR placebos OR placebo effect OR placebo response OR nocebo OR nocebos) AND (Depression OR depressive OR Anxiety OR anxious OR major depressive disorder OR MDD OR generalized anxiety disorder OR GAD OR panic disorder OR simple phobia OR antidepressant OR fluoxetine OR Citalopram OR Escitalopram OR Fluvoxamine OR Paroxetine OR Sertraline OR anxiolytic OR anxiolytics OR benzodiazepine OR benzodiazepines OR alprazolam OR clonazepam OR diazepam OR lorazepam OR temazepam) AND (positron emission tomography OR PET OR Single-photon emission computed tomography OR SPECT OR functional magnetic resonance imaging OR functional MRI OR fMRI OR tomography OR cerebral blood flow)

Limits: **none**

Results: 3098 (+ 397 in updated search) **hits**

**WEB OF KNOWLEDGE**

**(Web of science (science citation index expanded), Biological abstracts, Biosis, Food science and technology abstracts)**

Search terms:

Placebo OR placebos OR placebo effect OR placebo response OR nocebo OR nocebos

and

Depression OR depressive OR Anxiety OR anxious OR major depressive disorder OR MDD OR generalized anxiety disorder OR GAD OR panic disorder OR simple phobia OR antidepressant OR fluoxetine OR Citalopram OR Escitalopram OR Fluvoxamine OR Paroxetine OR Sertraline OR anxiolytic OR anxiolytics OR benzodiazepine OR benzodiazepines OR alprazolam OR clonazepam OR diazepam OR lorazepam OR temazepam

and

positron emission tomography OR PET OR Single-photon emission computed tomography OR SPECT OR functional magnetic resonance imaging OR functional MRI OR fMRI OR tomography OR cerebral blood flow

Limits: **none**

Results: 1362 (+ 159 in updated search) **hits**

**AFTER MERGING AND PARTIALLY REMOVING DUPLICATES:**

**3,999 POTENTIAL REFERENCES TO SCREEN**
