# Supplementary material for: Functional Neuroimaging Correlates of Placebo Response in Patients With Depressive or Anxiety Disorders: A Systematic Review
Source: Int J Neuropsychopharmacol. 2022 Jan 25;25(6):433–47. doi: 10.1093/ijnp/pyac009 (PMC9211006; doi:10.1093/ijnp/pyac009)
Supplement: pyac009_suppl_Supplementary_Data [file pyac009_suppl_supplementary_data.pdf]

		Domain 1: Randomization Process						Domain 2: Deviations from intended interventions										Domain 3: Missing outcome data							Domain 4: Measurement of the outcome								Domain 5: Selection of the reported result						
Reference	Sources Reviewed	1.1	1.2	1.3	Algorithm Result	Assessor judgement	Comments	2.1	2.2	2.3	2.4	2.5	2.6	2.7	Algorithm Result	Assessor judgement	Comments	3.1	3.2	3.3	3.4	Algorithm Result	Assessor judgement	Comments	4.1	4.2	4.3	4.4	4.5	Algorithm Result	Assessor judgement	Comments	5.1	5.2	5.3	Algorithm Result	Assessor judgement	Comments	Overall judgement
Chin Fatt 2020	Journal article(s); Statistical analysis plan (SAP); Non-commercial trial registry record (e.g. ClinicalTrials.gov record)	PY	PY	N	Low	Low	No detailed description of allocation sequence, but large multi-centre study, authors have previous experience of RCTs with randomization. As per ROB2 tool, we have answered 'Probably Yes' to 1.1 and 1.2.	N	N	NA	NA	NA	Y	NA	Low	Low	Double-blind RCT. ITT analysis with some participants excluded after randomisation due to poor quality data. Domain 2.6 therefore coded as 'Yes'.	Y	NA	NA	NA	Low	Low		N	N	PN	NA	NA	Low	Low	Outcomes were pre-specified in published protocols. Main outcome was Hamilton rating scale, which is appropriate. The study was double-blind.	Y	N	N	Low	Low	Analysis plan was pre-specified, and analysis has been carried out as per plan.	Low risk
Chin Fatt 2021a	Journal article(s); Statistical analysis plan (SAP); Non-commercial trial registry record (e.g. ClinicalTrials.gov record)	PY	PY	N	Low	Low	No detailed description of allocation sequence, but large multi-centre study, authors have previous experience of RCTs with randomization. As per ROB2 tool, we have answered 'Probably Yes' to 1.1 and 1.2.	N	N	NA	NA	NA	Y	NA	Low	Low	Double-blind RCT. ITT analysis with some participants excluded after randomisation due to poor quality data. Domain 2.6 therefore coded as 'Yes'.	N	NA	NI	PN	Some concerns	Some concerns	Only 82% of participants had complete baseline data and were included (n=244). Unknown if missingness is related to subsequent treatment outcome, although this is probably unlikely.	N	N	PN	NA	NA	Low	Low	Outcomes were pre-specified in published protocols. Main outcome was Hamilton rating scale, which is appropriate. The study was double-blind.	Y	N	N	Low	Low	Analysis plan was pre-specified, and analysis has been carried out as per plan.	Some concerns
Chin Fatt 2021b	Journal article(s); Statistical analysis plan (SAP); Non-commercial trial registry record (e.g. ClinicalTrials.gov record)	PY	PY	N	Low	Low	No detailed description of allocation sequence, but large multi-centre study, authors have previous experience of RCTs with randomization. As per ROB2 tool, we have answered 'Probably Yes' to 1.1 and 1.2.	N	N	NA	NA	NA	Y	NA	Low	Low	Double-blind RCT. ITT analysis with some participants excluded after randomisation due to poor quality data. Domain 2.6 therefore coded as 'Yes'.	Y	NA	NA	NA	Low	Low		N	N	PN	NA	NA	Low	Low	Outcomes were pre-specified in published protocols. Main outcome was Hamilton rating scale, which is appropriate. The study was double-blind.	Y	N	N	Low	Low	Analysis plan was pre-specified, and analysis has been carried out as per plan.	Low risk
Cooper 2019	Journal article(s); Statistical analysis plan (SAP); Non-commercial trial registry record (e.g. ClinicalTrials.gov record)	PY	PY	N	Low	Low	No detailed description of allocation sequence, but large multi-centre study, authors have previous experience of RCTs with randomization. As per ROB2 tool, we have answered 'Probably Yes' to 1.1 and 1.2.	N	N	NA	NA	NA	Y	NA	Low	Low	Double-blind RCT. ITT analysis with some participants excluded after randomisation due to poor quality data. Domain 2.6 therefore coded as 'Yes'.	N	N	PN	NA	Low	Low	Only 78% of participants had usable ASL data. Missingness unlikely to depend on its true value.	N	N	PN	NA	NA	Low	Low	Outcomes were pre-specified in published protocols. Main outcome was Hamilton rating scale, which is appropriate. The study was double-blind.	Y	N	N	Low	Low	Analysis plan was pre-specified, and analysis has been carried out as per plan.	Low risk
Fan 2020	Journal article(s); Statistical analysis plan (SAP); Non-commercial trial registry record (e.g. ClinicalTrials.gov record)	PY	PY	NI	Low	Low	No detailed description of allocation sequence, sample comes from EMBARC studies and so we have continued to answer 'Probably Yes' to 1.1 and 1.2.	N	N	NA	NA	NA	Y	NA	Low	Low	Double-blind RCT. ITT analysis with some participants excluded after randomisation due to poor quality data. Domain 2.6 therefore coded as 'Yes'.	N	NA	NI	NI	High	High	This study appears to be from a separate group from the EMBARC consortium. Only 200/296 EMBARC patients included. No information regarding reasons for missing data.	N	N	PN	NA	NA	Low	Low	Outcome for depressive symptoms appropriate (Hamilton rating scale). Pre-registered EMBARC protocol describes study as double-blind.	NI	N	NI	Some concerns	Some concerns	This is an exploratory analysis that does not appear to have been pre-planned.	High risk
Faria 2012	Journal article(s); Non-commercial trial registry record (e.g. ClinicalTrials.gov record); Company-owned trial registry record (e.g. GSK Clinical Study Register record); Regulatory document (e.g. Clinical Study Report, Drug Approval Package)	PY	PY	N	Low	Low	No detailed description of allocation sequence, study reports a number of trials, all likely to have been appropriately randomised based on authors' track record. No differences between studies prior to pooling results.	PN	PN	NA	NA	NA	Y	NA	Low	Low	Double-blind RCT's with appropriate shams. Intention-to-treat analysis.	Y	NA	NA	NA	Low	Low	Less than 2% of data missing	N	N	PN	NA	NA	Low	Low	Data collection not inappropriate. Outcomes of LSAS and STAI-S. The RCT's were double-blind.	N	N	NI	Some concerns	Some concerns	It is not clear from sources examined whether it was planned to dichotomise the SSRI and placebo groups into responders and non-responders. Such dichotomisation can cause false positives. No pre-registered analysis plan available.	Some concerns
Faria 2014	Journal article(s); Non-commercial trial registry record (e.g. ClinicalTrials.gov record); Company-owned trial registry record (e.g. GSK Clinical Study Register record); Regulatory document (e.g. Clinical Study Report, Drug Approval Package)	PY	PY	N	Low	Low	No detailed description of allocation sequence, study reports a number of trials, all likely to have been appropriately randomised based on authors' track record. No differences between studies prior to pooling results.	PN	PN	NA	NA	NA	Y	NA	Low	Low	Double-blind RCT's with appropriate shams. Intention-to-treat analysis.	Y	NA	NA	NA	Low	Low	Less than 2% of data missing	N	N	PN	NA	NA	Low	Low	Data collection not inappropriate. Outcomes of LSAS and STAI-S. The RCT's were double-blind.	N	N	NI	Some concerns	Some concerns	It is not clear from sources examined whether there it was planned to dichotomise the SSRI and placebo groups into responders and non-responders. Such dichotomisation can cause false positives. No pre-registered analysis plan available.	Some concerns
Faria 2017	Journal article(s); Non-commercial trial registry record (e.g. ClinicalTrials.gov record)	Y	Y	N	Low	Low	Full description of allocation sequence and randomization procedures present	N	N	NA	NA	NA	Y	NA	Low	Low	The study was double-blind. Intention to treat analysis.	Y	NA	NA	NA	Low	Low	Only 1 participant dropped out, equates to 2% of data	N	N	N	NA	NA	Low	Low	Data collection not inappropriate. RCT was double-blind.	NI	N	NI	Some concerns	Some concerns	Trial was retrospectively registered. No evidence of a pre-registered analysis plan.	Some concerns
Furmark 2008	Journal article(s)	NI	PY	N	Low	Low	Article reports that studies were 'randomised'. Also double-blind. Likely that allocation sequence was concealed based on authors' track record.	N	N	NA	NA	NA	Y	NA	Low	Low	Double-blind studies with appropriate shams.	Y	NA	NA	NA	Low	Low	Re-analysis of previously collected trial data	N	N	N	NA	NA	Low	Low	Appropriate outcome measures used. Studies were double-blind.	NI	NI	NI	Some concerns	Some concerns	Re-analysis of previously collected trial data. No evidence of a pre-registered plan. Unclear whether this re-analysis was planned. If not then risk of false positives due to small sample size.	Some concerns
Greenberg 2020	Journal article(s); Statistical analysis plan (SAP); Non-commercial trial registry record (e.g. ClinicalTrials.gov record)	PY	PY	N	Low	Low	No detailed description of allocation sequence, but large multi-centre study, authors have previous experience of RCTs with randomization. As per ROB2 tool, we have answered 'Probably Yes' to 1.1 and 1.2.	N	N	NA	NA	NA	Y	NA	Low	Low	Double-blind RCT. ITT analysis with some participants excluded after randomisation due to poor quality data. Domain 2.6 therefore coded as 'Yes'.	N	PN	PN	NA	Low	Low	Only 75% of participants had baseline reward imaging data. Unlikely missingness depended on its true value.	N	N	PN	NA	NA	Low	Low	Outcomes were pre-specified in published protocols. Main outcome was Hamilton rating scale, which is appropriate. The study was double-blind.	Y	N	N	Low	Low	Analysis plan was pre-specified, and analysis has been carried out as per plan.	Low risk
Mayberg 2002	Journal article(s)	NI	PY	N	Low	Low	Study reported as 'randomised' with no further details. Also reported as double-blind, so allocation sequence was probably concealed.	PN	PN	NA	NA	NA	Y	NA	Low	Low	Trial reported as double-blind. No evidence participants were aware of assigned intervention. Intention-to-treat analysis.	N	N	PN	NA	Low	Low	Approximately 10% of participants could not be scanned due to mechanical issues. Unlikely this missingness depended on its true value.	N	N	N	NA	NA	Low	Low	Outcome measurement not inappropriate (Hamilton depression rating scale). Study was double-blind.	NI	NI	NI	Some concerns	Some concerns	No evidence of a pre-specified analysis plan. Not enough information to make assessments about domains 5.2 and 5.3.	Some concerns
Pecina 2015	Journal article(s); Non-commercial trial registry record (e.g. ClinicalTrials.gov record)	NI	NI	N	Some concerns	Some concerns	Study reported as 'randomised' only with no further details. Unclear whether investigators were blinded to treatment assignment.	Y	Y	N	NA	NA	Y	NA	Low	Low	Participants aware of intervention (i.e. active or inactive) during placebo lead-in phase as the manipulation required this. Second phase was open-label. There was a modified ITT analysis excluding participants with missing outcome data.	N	N	N	NA	Low	Low	Approximately 30% of patients dropped out post-randomisation. No sensitivity analysis (e.g. involving imputation) to assess impact of this. Reasons for discontinuation ('reluctance to take medication', 'long distance to appointments', 'small compensation') likely not related to outcome value.	N	N	Y	PN	NA	Low	Low	Outcome measures for depression (QIDS) and PET appropriate. Outcome assessors were likely aware of participants' assignment due to differing instructions given in placebo lead-in phase and second phase was open-label. This was unlikely to affect outcome measure as QIDS is self-report.	NI	NI	NI	Some concerns	Some concerns	Unable to find a pre-registered analysis plan. Analysis restricted to a priori regions of interest with no whole brain analysis. Due to lack of a pre-registered analysis plan we are unable to make a judgement about whether this represents selective reporting.	Some concerns
Pecina 2018	Journal article(s)	Y	PY	N	Low	Low	A trial-by-trial manipulation of expectancies. Participants randomly shown high/low expectancy and infusion/no infusion cues.	Y	N	N	NA	NA	Y	NA	Low	Low	Participants were aware of the meaning of the cues, this was important for the study design. They were unaware that the neurofeedback was sham.	Y	NA	NA	NA	Low	Low	No evidence of missing data	N	N	Y	PN	NA	Low	Low	The study was double-blind. Participants self-rated mood and expectancy.	NI	NI	PN	Some concerns	Some concerns	No pre-registered analysis plan. However, there does not appear to have been selective reporting.	Some concerns
Pecina 2021	Journal article(s)	Y	Y	N	Low	Low	Randomisation list was generated by a group of independent pharmacists, and this was concealed from investigators.	N	N	NA	NA	NA	Y	NA	Low	Low	The study was double-blind. Participants were unaware of whether they received naltrexone or placebo.	N	N	PN	NA	Low	Low	20% of patients dropped out or were excluded post-randomisation due to claustrophobia, positive pregnancy test, or missing outcome data. Missingness unlikely related to its true value.	N	N	PN	NA	NA	Low	Low	The study was double-blind. Participants self-rated mood and expectancy.	NI	N	N	Some concerns	Some concerns	No evidence of a pre-specified analysis plan. However, fMRI analysis pipeline and behavioural analyses are similar to Pecina 2018.	Some concerns

Sikora 2016	Journal article(s); Non-commercial trial registry record (e.g. ClinicalTrials.gov record)	NI	NI	N	Some concerns	Some concerns	Study reported as 'randomised'. Unclear whether investigators were blinded to treatment assignment.	Y	Y	N	NA	NA	Y	NA	Low	Low	Participants aware of intervention (i.e. active or inactive) during placebo lead-in phase as the manipulation required this. Second phase was open-label. There was a modified ITT analysis excluding participants with missing outcome data.	N	Y	NA	NA	Low	Some concerns	Approximately 20% of patients did not complete study. Supplementary data shows no difference in non-completers compared with completers in depression symptoms and placebo responsiveness. However, this sample only includes 29 of the 35 patients from Pecina 2015. Both have same clinicaltrials.gov entry. It is not clear why the other patients' data is not reported/collected.	N	N	Y	PN	NA	Low	Low	Outcome measures for depression (QIDS) and fMRI appropriate. Outcome assessors were likely aware of participants' assignment due to differing instructions given in placebo lead-in phase and second phase was open-label. This was unlikely to affect outcome measure as QIDS is self-report.	NI	NI	NI	Some concerns	Some concerns	Unable to find a pre-registered analysis plan. Small volume correction appears to have been used to constrain results within networks of interest. Due to lack of a pre-registered analysis plan unable to make a judgement about whether this represents selective reporting.	Some concerns
Zilcha-Mano 2019	Journal article(s); Non-commercial trial registry record (e.g. ClinicalTrials.gov record)	NI	PY	N	Low	Low	Study reported as 'randomised'. Efforts appear to have been made to conceal allocation sequence, "neither participants nor outcome assessors were aware of the 4:1 randomization schedule or the specific treatment assignment to medication or placebo".	Y	PY	N	NA	NA	Y	NA	Low	Low	Participants were aware of their intervention as this was an important manipulation of expectancy in the trial. ITT analysis.	Y	NA	NA	NA	Low	Low	No evidence of missing data. No significant differences in demographic or clinical measures between those who were and were not scanned.	N	N	N	NA	NA	Low	Low	Outcome assessors were blinded to group assignment. Appropriate measure of depressive symptoms (Hamilton rating scale).	NI	N	Y	High	High	No pre-registered analysis plan, although outcome measures appear to have been described a priori on clinicaltrials.gov record. However, some evidence of selective reporting. Only one task is described despite 3 tasks being listed on clinicaltrials.gov. The amygdala only showed a significant difference in activity in the sad vs. neutral faces contrast, whereas regions such as dorsolateral prefrontal cortex demonstrated significant differences in activity in other relevant contrasts. The amygdala is then chosen as a region of interest with little justification, and no further analyses regarding other significant regions are reported	High risk
